# Reaching movements are attracted by stimuli that signal reward

**DOI:** 10.3758/s13414-020-02120-0

**Published:** 2020-09-10

**Authors:** Tom Nissens, Katja Fiehler

**Affiliations:** Experimental Psychology, Justus-Liebig University Giessen, Otto-Behaghel-Strasse 10F, 35394 Giessen, Germany

**Keywords:** Goal-directed movements, Perception and action, Visual search

## Abstract

When presented with a set of possible reach targets, the movement trajectory can reveal aspects of the underlying competition for action selection. Current goals and physical salience can affect the trajectory of reaching movements to be attracted towards a distractor. Some studies demonstrated that stimuli associated with reward can also cause an attraction when reaching towards the reward stimulus was previously rewarded and the reward stimulus was physically salient. Here we demonstrate that a non-salient stimulus that signals the availability of reward attracts reaching movements even when moving towards it was never necessary nor rewarded. Moreover, the attraction by reward is particularly evident with short-latency movements. We conclude that neither physical salience nor reinforcing the movement towards a stimulus is necessary for reward to gain priority in the selection for action.

## Introduction

Movements towards a target can be influenced by the current goals of the observer (Chapman et al., 2014; Moehler & Fiehler, 2017; Nissens & Fiehler, 2018) and the physical salience of the stimuli (Cisek & Kalaska, 2010; Gallivan, Chapman, Wolpert, & Flanagan, 2018; Herwig, 2015; Moher, Anderson & Song, 2015; Schneider, Einhauser, & Horstmann, 2013; Song, 2017; Wispinski, Gallivan, & Chapman, 2020). For example, the movement trajectory is often attracted towards a physically salient distractor (Nissens & Fiehler, 2020; Welsh, Elliott, & Weeks, 1999). This has been explained by competing movement plans during target selection. The movement plan towards the distractor competes with the plan towards the target for action selection. When the competition is not resolved when the reach is initiated, the initial direction of the movement deviates towards the distractor. Consequently, the plan towards the distractor is further suppressed and the movement direction adjusted so that the reach ends at the target.

Apart from the current goals and physical salience, stimuli associated with reward can also influence movement trajectories (Chapman, Gallivan, & Enns, 2015a; Chapman, Gallivan, Wong, Wispinski, & Enns, 2015b; Moher, Anderson & Song, 2015; Wirth, Dignath, Pfister, Kunde & Eder, 2016). In previous studies, participants received reward after reaching to a target in a certain color that established an association between that color and reward. On subsequent trials in the same (Chapman et al., 2015a, 2015b; Wirth et al., 2016) or different (Chapman et al., 2015a, 2015b; Moher et al., 2015) blocks the rewarded color functioned as a distractor. They found that the reaching trajectory was attracted towards the reward-associated distractor. Two points are left open: (i) As reaching towards the reward color was reinforced, it is unknown if the attraction by the reward distractor is due to a learned association between the color and reward or the action of reaching towards the color and reward. This raises the question whether the color itself or the reaching towards the color gains priority. (ii) As the reward-associated distractor was physically salient (Moher et al., 2015) or presented with only one alternative shape (Chapman et al., 2015a, 2015b; Wirth et al., 2016), it is unknown if a reward distractor would attract reaching movements if it was not physically salient, i.e. presented among multiple differently colored shapes.

There is a growing body of literature showing that stimuli associated with reward attract visual attention and eye movements (Anderson, 2016; Chelazzi, Perlato, Santandrea, & Della Libera, 2013; Failing & Theeuwes, 2018). For example, Failing et al. (2015) showed that when participants had to fixate a shape singleton, a non-salient distractor captured the eyes more often when it signaled high compared to low reward. Moreover, this effect was more pronounced in very early saccades. In the current study, we use a similar design to uncover the influence of a reward-signaling distractor on reaching movements.

Here, we investigated whether a reward-signaling stimulus that was never the target and not physically salient can influence reaching movements towards a target. Participants reached towards a target square presented at one of four possible target locations. The remaining three locations were occupied by differently colored distractor circles of which one could be in a reward-signaling color. We expected the reaching movement to be attracted towards the non-physically salient reward-signaling distractor driven by an association between color and reward. Since reaching towards the reward-signaling color was never rewarded nor necessary, we can rule out an attraction due to an action-reward association. Furthermore, given that the reward-signaling distractor is not physically salient, we can also rule out an initial attraction by saliency that is magnified by reward. Following the model by Chapman et al. (2015b), we also expected the attraction by reward to be more pronounced on short compared to long latency reaches.

## Method

### Participants

Twenty-seven volunteers with reported normal or corrected-to-normal vision participated in the experiment. Four participants were excluded due to less than 50% of trials meeting the inclusion criteria in one of the conditions (see *Analyses*), two participants due to data corruption and one participant for misunderstanding the instructions. This resulted in a final sample of 20 participants (11 males, mean age 25 years). The sample size was estimated using GPower (Faul, Erdfelder, Lang, & Buchner, 2007) based on the effect size of reach curvature observed in a pilot study, n = 4, *d*_*z*_ = 0.87, with *α* error probability = 0.05, and power = 0.95. This resulted in a sample size estimate of 20 participants. All participants were right-handed according to the Edinburgh Handedness Inventory (M = 78.8, SD = 18.0; Oldfield, 1971). Participants gave written informed consent prior to the experiment and received course credits or financial compensation. In addition, collected reward points were paid out in money (M = 5.25€, SD = 0.47€). The study was approved by the Giessen University ethics committee and was conducted in accordance with the Declaration of Helsinki (2008).

### Apparatus

Stimuli were created with the Psychophysics Toolbox (Kleiner et al., 2007) in Matlab and presented on a vertically angled, VIEWPixx monitor (1,920 x 1,200 pixels, 120 Hz). Reach movements were recorded with an optoelectronic motion-tracking system (Optotrak Certus), which registered an infrared marker placed on the fingernail of the right index finger with a sampling rate of 250 Hz. Monocular movements of participants’ right eyes were recorded via a head-mounted video-based EyeLink II with a sampling rate of 500 Hz. Participants’ heads were positioned on a chin rest at a distance of 48 cm from the screen. The experiment was performed in the dark.

### Stimuli

**Fig. 1:**
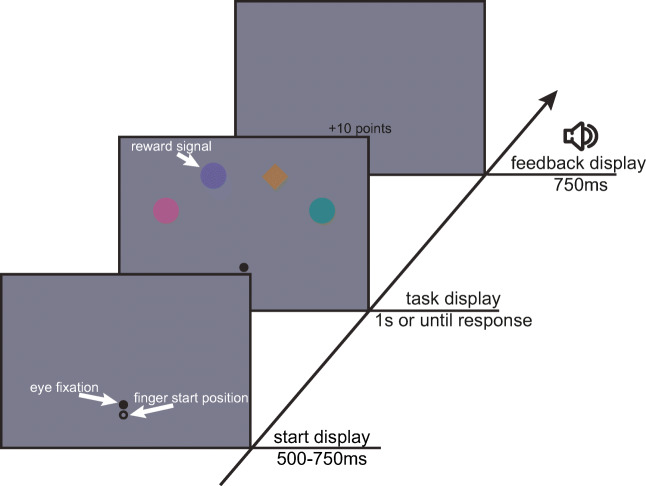
Sequence of trial events. Participants had to reach to a diamond shape presented among circles. In some trials one of the distractor circles was in a color that signaled the possibility of earning either high or low reward on that trial

The start display consisted of two black circles (0.42 vd (visual degrees radius)) on a gray background. An eye-fixation circle presented 2.5 vd below screen center and a finger-start circle presented 1.5 vd below the eye fixation circle (Fig. 1). In the task display, the finger start circle was removed and four shapes (1.25 vd, 11 mm radius), comprising one target diamond and three distractor circles, were positioned on an imaginary arc (10 vd, 88 mm radius) around eye fixation with 36 angular degrees between neighboring shapes. All shapes were uniquely colored. During the feedback display, written feedback was presented at the location of the eye-fixation circle, together with the presentation of a high or low auditory beep denoting correct or incorrect performance, respectively.

All stimulus colors were defined in LAB color space (CIE, 1967) consisting of three coordinates: Lightness (≈luminance), A (green-red axis), and B (blue-yellow axis). The background (LAB: 50,0,0) and the shapes were isoluminant. The six shape colors had equal chroma, and were equally spaced around the LAB color space (LAB coordinates of the six shape colors: 50, 23.5, 40.7; 50, -23.5, 40.7; 50, 47, 0; 50, -23.5, -40.7; 50, 23.5, -40.7; 50, 47, 0). For each participant, one of these colors was randomly chosen as the high reward-signaling color and another one as the low reward-signaling color. On high- and low-reward trials one of the distractors was in the high- or low-reward-signaling color, respectively. The colors of the other shapes were randomly sampled from the remaining set of colors without replacement. On baseline trials the colors of all shapes were sampled from the remaining set of colors.

### Procedure

The fixation/start screen was presented for a randomized minimum time of 500 ms or 750 ms, or until gaze and finger position were evaluated positively (finger within 5 mm in the x, y dimension and within 3 mm in z dimension from the center of the start circle). Next, the search display was presented for 1,000 ms or when a reach end was detected (finger velocity dropped below 20 mm/s after moving more than 40 mm from the start position). Only when the reach ended before a variable deadline and within 28 mm from the center of the target shape, was the trial evaluated as correct. The variable deadline was based on the 80th percentile of the response times (reach latency + movement time) of the last 100 trials in which participants reached the target before the offset of the search display, i.e. within 1,000 ms. Before 100 trials were gathered, the variable deadline was set to 700 ms based on another study from our group with a similar design (Nissens & Fiehler, 2020). Next, the feedback display was presented for 750 ms, indicating how much reward was earned on that trial (+10, +1, or 0 points) together with an auditory beep (high pitch for correct; low pitch for incorrect). Participants earned 10 points for correct responses on high-reward trials, 1 point for correct responses on low-reward trials, and 0 points for correct responses on baseline trials and incorrect responses on any trial type. In other words, participants did not earn reward on baseline trials, and on high- and low-reward trials where they did not reach the target before the variable deadline. During practice blocks, the feedback display denoted information about the performance on that trial (correct, too slow, eyes moved, wrong shape, or correct but too slow).

Participants were asked to reach as quickly and accurately as possible to the diamond shape while maintaining fixation. During practice, only baseline trials were presented, i.e. the reward-signaling colors were not presented. After practice, participants were informed that they could now earn reward depending on their performance and that there are high- and low-reward-signaling colors associated with 10 or 1 points, respectively. It was explicitly stated that the target would never be in one of these colors, thus had to be ignored. Also, participants were informed that they would only earn reward if they reached the target before a deadline and did not make an eye movement.

Each participant performed 40 practice trials and 792 experimental trials divided into eight blocks. All possible combinations (36) of target position (4), distractor position relative to the target (3) and distractor condition (3) were counterbalanced; resulting in 22 repetitions of each combination.

### Analyses

Small sections (M = 4 samples, SD = 5 samples) of missing reach data due to the temporarily blocked view of the marker were interpolated for each dimension separately. In the offline analysis, the starting point of a reach was defined as the first sample of four consecutive vector velocity readings greater than 25 mm/s where there was a total acceleration of 20 mm/s^2^ across the four points. The endpoint of a reach was defined as the point in time when the velocity dropped below 20 mm/s (Chapman & Goodale, 2010). Reaching movements were normalized by resampling each movement to 101 samples equally spaced along the reach amplitude. Saccades start- and endpoints were detected online using minimum velocity and acceleration criteria of 30 vd/s and 8,000 vd/s^2^, respectively.

Trials were excluded when at least one of the following criteria was reached: a saccade of >2.5 vd was detected; the reach end was more than 28 mm away from the target center; the reach start was more than 10 mm from the finger start circle; the maximum reach velocity was >5,000 mm/s; the reach latency was <200 ms or >600 ms. Over all criteria and all participants 9.72% of trials were excluded.

To determine whether reaching movements deviated towards or away from the reward-signaling distractor, we calculated the attraction score (Moher et al., 2015). The attraction score denotes the distance at a certain point along the trajectory between the baseline condition and one of the reward-signaling distractor conditions relative to the reward-signaling distractor’s location, with positive values indicating deviation towards, i.e. attraction, and negative values indicating deviation away. Reach curvature is the average of the attraction score values.

To determine when the distractor attracted the reaching movement, we performed a cluster-based analysis (Maris & Oostenveld, 2007; Moher et al., 2015). The *t*-statistics for the distractor attraction score were calculated for each point along the normalized space, then the largest cluster of consecutive t-values for which *p* < 0.05 was detected, and the sum of the *t*-values in that cluster were calculated. If the observed cluster size was significant with *p* < 0.05 under the estimated probability density function, the portion of the reaching movement related to this cluster is reported to be affected by the distractor.

To further investigate the time course of the effect of reward on reaching movements, the attraction score and reach curvature was calculated for movements with short and long latencies, separately. Moreover, a median split based on reach latency was performed for each participant, each distractor condition and each combination of target and distractor location, separately. The mean latency was 298 ms (SD = 34 ms) for short and 366 ms (SD = 43 ms) for long latency bins.

## Results

### Reaching movement curvature

**Fig. 2:**
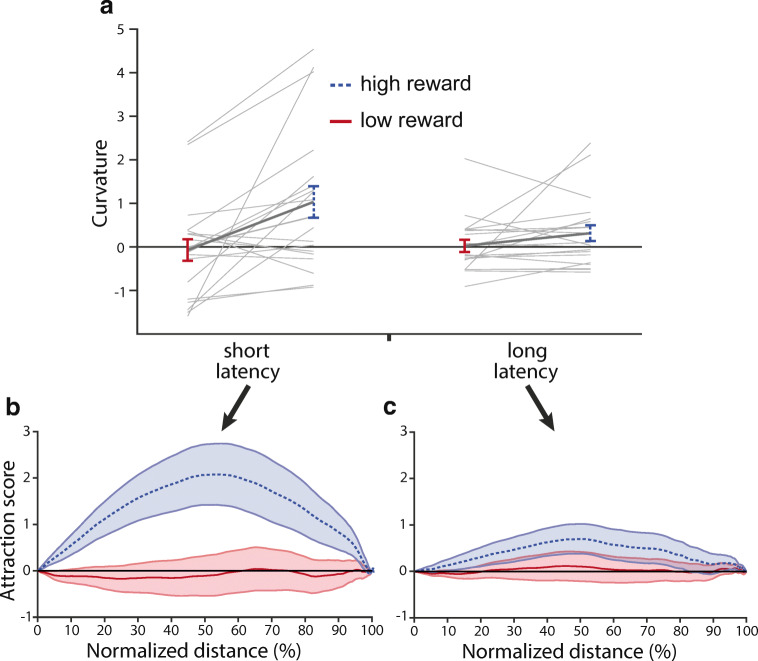
(**A**) The magnitude of reach curvature by reward-signaling value of the distractor separately for short-latency trials (left) and long latency trials (right). Positive values denote curvature towards the distractor. Distractor attraction scores along the normalized movement amplitude by reward-signaling value of the distractor for short-latency trials (**B**) and long-latency trials (**C**). Positive values denote attraction towards the distractor. All error bars reflect SEM

To investigate whether a high-reward-signaling stimulus, that is physically non-salient and never a target, influences the curvature of reaching movements to a searched target (Fig. 2A), we performed a within-subjects analysis of variance (ANOVA) with the factors reward (low vs. high) and reach latency (short vs. long). There was a main effect of reward, *F*(1,19) = 8.461, *p* = 0.009, *η*_*p*_^*2*^ = 0.308, which showed that reaching movements curved more towards the high- compared to the low-reward-signaling distractor. There was no main effect of reach latency, *F*(1,19) = 2.252, *p* = 0.15, *η*_*p*_^*2*^ = 0.106. However, the interaction effect between reward and reach latency was significant, *F*(1,19) = 10.455, *p* = 0.004, *η*_*p*_^*2*^ = 0.355, which showed that the effect of reward on reach curvature was more pronounced for short (Fig. 2B) compared to long (Fig. 2C) reach latencies. Hence, reaches curved more towards the high- compared to the low-reward-signaling distractor and this even more so on short compared to long reach latencies. Next, we examined if the curvature on high- and low-reward trials differed from the curvature on baseline trials. Paired samples *t*-tests showed that reach curvature on high-reward trials was significantly different from baseline, *t*(19) = 2.698, *p* = 0.014, *d* = 0.603; whereas curvature on low-reward trials did not differ from baseline, *t*(19) = 0.128, *p* = 0.899, *d* = 0.029. Hence, reaches curved towards the high-, but not low-, reward-signaling distractor compared to baseline. We further tested whether reach curvature differed between short latency high-reward trials and long latency high-reward trials. We found that reach curvature on high-reward trials was more pronounced for short compared to long reach latencies, *t*(19) = 2.575, *p* = 0.019, *d* = 0.576. Furthermore, for long reach latencies, curvature was not different between high reward and baseline trials, *t*(19) = 1.739, *p* = 0.098, *d* = 0.389.

Next, we investigated whether participants whose latency was shorter showed a larger influence of reward on curvature. We ran a between-subjects Pearson correlation between reach latency and the effect of reward on curvature (high reward – low reward). We found that the effect of reward was larger for participants with shorter reach latencies; *r*(18) = -0.573, *p* = 0.008.

### Reaching movement trajectory and attraction score

To investigate where along the trajectory the reaching movement curved more towards the high- compared to low-reward-signaling distractors, we performed a cluster-based analysis on the attraction scores (see *Analyses*). The results show that the high-reward-signaling distractor attracted the reaching movement more from movement onset until 71% along the trajectory than the low-reward-signaling distractor. For short-latency movements (Fig. 2B), the reach was more attracted by the high- compared to the low-reward-signaling distractor from movement onset until 81% along the trajectory. However, for long-latency movements (Fig. 2C) there was no difference between the attraction scores for trials with a high- or low-reward-signaling distractor.

### Movement parameters

To investigate whether movement latency, movement time, and reaching endpoint accuracy were affected by reward, we ran one-way ANOVAs with the factor reward (low, high, and baseline). The effect of reward was not significant for movement latency, *F*(2,38) = 0.206, *p* = 0.814, *η*_*p*_^*2*^ = 0.011; movement time, *F*(2,38) = 3.115, *p* = 0.056, *η*_*p*_^*2*^ = 0.141; nor reaching endpoint accuracy, *F*(2,38) = 1.873, *p* = 0.168, *η*_*p*_^*2*^ = 0.090. For completeness, we ran paired-samples *t*-tests to investigate the trending effect of reward on movement time. Movement time was significantly different between low- (305 ms) and high- (309 ms) reward trials, *t*(19) = 2.415, *p* = 0.026, *d* = 0.540; but not between baseline (305ms) and low-reward trials, *t*(19) = 0.304, *p* = 0.764, *d* = 0.068; or baseline and high-reward trials, *t*(19) = 1.740, *p* = 0.098, *d* = 0.389.

## Discussion

The current study shows that a physically non-salient stimulus signaling high in contrast to low reward attracts reaching movements. Reaching movements clearly deviated towards the high-reward-signaling distractor even though it was never the reach target and reaching towards it was never rewarded. Conversely, reaching towards the distractor would have led to the omission of reward. The attraction by the high-reward-signaling distractor was present in short- but not in long-latency movements. The attraction of the reaching movement can be attributed to the reward-signaling stimuli obtaining an increased selection priority for action independently of physical salience. The priority for selection increases the likelihood of the activation of a movement plan towards the high-reward-signaling distractor. The activation of the movement plan causes the subsequent reaching movement to be deviated towards the high-reward-signaling distractor if the competition with the movement plan is not resolved. The competition is then resolved by suppressing the movement plan to the distractor. As this suppression increases gradually, attraction by the reward-signaling distractor is more pronounced for short than long-latency movements.

The current findings advance our understanding of how reward association can shape the selection for action. In previous studies (Chapman et al., 2015a, 2015b; Moher et al., 2015; Wirth et al., 2016), reaching to the stimulus associated with reward was previously rewarded, and thus reinforced. Therefore, it was unclear whether reinforcement learning of moving towards a reward-associated stimulus is necessary to observe consequent attraction when that stimulus is presented as a distractor. Moreover, the reward-associated distractor was always physically salient or presented with only one alternative shape, hence, it was unclear whether reward can gain priority in the selection for action when it is not physically salient. The current results can close these research gaps by showing that a non-salient stimulus associated with reward can gain priority in the selection for action, even when reaching to the reward-associated stimulus was neither necessary nor rewarded.

Moher et al. (2015) observed that reaching movements were less attracted towards a color previously associated with high compared to low reward. This is in contrast to our results showing more attraction towards the high- compared to the low-reward-signaling color. Although we cannot reconcile these seemingly opposing findings with our study, we would like to list several differences in the design of the two studies that might explain the different results. (i) In Moher’s study the target was presented in the reward color in the training phase, whereas we never displayed the target in the reward color. (ii) During experimental blocks, where the reward color was presented as a distractor, participants could not earn reward in Moher’s study whereas participants could earn reward in the present study. (iii) The reward color was physically salient when presented as a distractor in Moher’s study but not in the present study. (iv) The target was a unique shape in both studies but could be a diamond or circle in Moher’s study, whereas the target was always a diamond in the present study. (v) In Moher’s study the shapes were presented around fixation, whereas in the present study the shapes were presented above fixation. (vi) And finally, the reach start position was on a block placed in front of the screen on a table in Moher’s study, and directly on the screen in the present study.

The current findings show similarities with studies on the influence of reward-associated stimuli on visual selection. Failing et al. (2015) found that saccades are more often directed towards a non-salient reward-signaling distractor (see also Le Pelley, Pearson, Griffiths, & Beesley, 2015). Similarly, we found that the direction of reaching movements deviates towards a non-salient reward-signaling distractor. Also, the attraction by the reward-signaling distractor was mainly pronounced for short-latency-reaching movements, as was found for short-latency eye movements (Failing et al., 2015) and predicted by the model of Chapman et al. (2015b). In covert visual search tasks, where participants have to detect the orientation of a line segment within the target shape without making eye movements, reaction times are increased when a reward-signaling distractor is present (Le Pelley et al., 2015), and even when it is non-salient (Failing & Theeuwes, 2017). These results suggest that reward-signaling stimuli are prioritized during covert and overt visual selection and do affect visual selection and selection for action in a similar fashion.

The prioritization of reward-signaling stimuli during visual selection has been argued to be strategic, to gain information and reduce uncertainty about the possible outcome, rather than an involuntary attraction driven by reward value (Le Pelley, Mittchel, Beesley, George, & Wills, 2016; Watson, Pearson, Wiers, & Le Pelley, 2019). A behavioral study in non-human primates found an increase in oculomotor exploration to decrease uncertainty about the reward outcome, even though the exploration did not change the reward outcome (Daddaoua, Lopes, & Gottlieb, 2016). In the current study, reaching towards the reward-signaling distractor was never necessary, nor useful to obtain reward. Still, the reward-signaling distractor provides information about the possible reward outcome, and thus decreases outcome uncertainty. However, we argue that a strategic uncertainty reduction is not the driving factor here: (i) The attraction of reaches towards high-reward-signaling distractors was mainly pronounced on short-latency movements. Hence, the attraction seems to be an involuntary rather than a strategic selection or exploration to decrease outcome uncertainty. (ii) Both the low- and high-reward-signaling distractors offer the same amount of information and uncertainty reduction regarding the possible outcome. However, reaching movements were attracted more towards the high- compared to the low-reward-signaling distractor, suggesting that the reward value itself seems to be important, rather than the reduction of uncertainty. On a different note, even though the physical features of the reward-signaling distractor, shape and color, were never the target features, they were presented at potential target locations. Previous studies showed that reaches deviate away from task-relevant or task-irrelevant physically salient distractors that were never presented at a possible target location (Howard & Tipper, 1997; Moehler & Fiehler, 2017). Future research should address the question whether presenting the distractor at a possible target location is a determining factor in attracting reaches towards reward-associated stimuli.

Together, our findings show that a reward-associated stimulus gains priority in the action selection process, even when it is not physically salient, is never a movement target, and reaching towards it is never rewarded.
